# Correction: Lack of variations in the salamander chytrid fungus, *Batrachochytrium salamandrivorans*, at its alleged origin: Updating its Japanese distribution with new evidence

**DOI:** 10.1371/journal.pone.0333394

**Published:** 2025-09-26

**Authors:** David Lastra González, Kanto Nishikawa, Koshiro Eto, Shigeharu Terui, Ryo Kamimura, Nuria Viñuela Rodríguez, Natsuhiko Yoshikawa, Atsushi Tominaga

David Lastra González and Atsushi Tominaga, should be noted as shared co-corresponding authors. Atsushi Tominaga’s email address is: tominaga@cs.u-ryukyu.ac.jp.
