# Supplementary material for: Lack of variations in the salamander chytrid fungus, Batrachochytrium salamandrivorans, at its alleged origin: Updating its Japanese distribution with new evidence
Source: PLoS One. 2024 Jun 13;19(6):e0305228. doi: 10.1371/journal.pone.0305228 (PMC11175521; doi:10.1371/journal.pone.0305228)
Supplement: S1 Table — *Bsal+ detected by Blooi et al. 2013 (less 40 Ct value, duplicate, sigmoidal curve). †Bsal+ detected by Blooi et al. 2013 (more qPCR positives than negatives as Spitzen-van der Sluijs et al. 2020). ‡Bsal+ sequenced by Sanger and 100% identical amplicon as Genbank reference accession number KC762295. (DOCX) [file pone.0305228.s001.docx]

**Supplementary Information for**

**Full title: Lack of variations in the salamander chytrid fungus, Batrachochytrium salamandrivorans, at its alleged origin: Updating its Japanese distribution with new evidence.**

**Short title: Updating *Batrachochytrium salamandrivorans* in Japan.**

David Lastra González^1,2,*^, Kanto Nishikawa^3^, Koshiro Eto^4^, Shigeharu Terui ^5^, Ryo Kamimura^6^ Nuria Viñuela Rodríguez^7^, Natsuhiko Yoshikawa^8^ & Atsushi Tominaga^1,6^

David Lastra González, JSPS International Research Fellow, ^1^Department of Natural Sciences, Faculty of Education, University of the Ryukyus, Nishihara, Okinawa, Japan

^2^Department of Ecology, Faculty of Environmental Sciences, Czech University of Life Sciences Prague, Prague – Suchdol, Czech Republic

Kanto Nishikawa, ^3^Graduate School of Global Environmental Studies, Kyoto University, Yoshida Hon-machi, Sakyo, Japan

Koshiro Eto, ^4^Kitakyushu Museum of Natural History & Human History, Kitakyushu, Fukuoka, Japan

Shigeharu Terui, ^5^Environment Grasp Promotion Network-PEG, Nonprofit Organization, Kushiro-shi, Hokkaido, Japan

Ryo Kamimura, ^6^Graduate school of Engineering and Science, University of the Ryukyus, Nishihara, Okinawa, Japan

Nuria Viñuela Rodríguez, ^7^Molecular Invertebrate Systematics and Ecology [MISE] Lab, Graduate school of Engineering and Science, University of the Ryukyus, Nishihara, Okinawa, Japan

Natsuhiko Yoshikawa, ^8^Department of Zoology, National Museum of Nature and Science, Tokyo, Tsukuba, Ibaraki, Japan

Atsushi Tominaga, ^1^Department of Natural Sciences, Faculty of Education, University of the Ryukyus, Nishihara, Okinawa, Japan

^6^Graduate school of Engineering and Science, University of the Ryukyus, Nishihara, Okinawa, Japan

Corresponding author: [g222001@edu.u-ryukyu.ac.jp](mailto:g222001@edu.u-ryukyu.ac.jp) (DLG)

S1 Table. Detailed information about amphibian collected samples.

*Bsal+ detected by qPCR as Blooi et al. 2013 (less 40 Ct value, duplicate, sigmoidal curve).

†Bsal+ detected by qPCR as Blooi et al. 2013 (Bsal sample is considered positive if during qPCR analyses more qPCR replicates became positives than negatives as Spitzen-van der Sluijs et al. 2020, 18).

‡Bsal+ sequenced by Sanger and 100% identical amplicon as Genbank reference accession number KC762295.

| Island | Location | Species | Year | N | Bsal+* | Bsal+^†^ | Bsal+^‡^ | Bd+ |
| --- | --- | --- | --- | --- | --- | --- | --- | --- |
| Okinawa | Nakayama, Nanjo | *Cynops ensicauda* | 2022 | 20 | 2 | 1 | 2 | 5 |
| Okinawa | Kitanakagusuku | *Cynops ensicauda* | 2022 | 20 |  | 2 | 1 | 5 |
| Okinawa | Onna | *Cynops ensicauda* | 2022 | 9 | 2 |  | 2 | 2 |
| Okinawa | Yonna, Kunigami | *Cynops ensicauda* | 2023 | 16 |  | 1 | 1 | 4 |
| Okinawa | Ogimi | *Cynops ensicauda* | 2023 | 20 |  | 1 | 1 | 11 |
| Okinawa | Kitanakagusuku | *Cynops ensicauda* | 2023 | 9 |  |  |  | 1 |
| Okinawa | Kudeken, Nanjo | *Cynops ensicauda* | 2023 | 20 |  |  |  |  |
| Tokunoshima | Tokunoshima | *Echinotriton raffaellii* | 2023 | 21 |  |  |  |  |
| Tokunoshima | Tomori, Amagi | *Echinotriton raffaellii* | 2023 | 3 |  |  |  |  |
| Amami | Ohama, Amami | *Cynops ensicauda* | 2023 | 20 |  |  |  |  |
| Amami | Tatsugo | *Cynops ensicauda* | 2023 | 21 |  |  |  |  |
| Amami | Itsubugach, Amami | *Cynops ensicauda* | 2023 | 21 |  |  |  |  |
| Amami | Yuwan-1, Yamato | *Cynops ensicauda* | 2023 | 20 |  |  |  |  |
| Amami | Yuwan-2, Yamato | *Cynops ensicauda* | 2023 | 11 |  |  |  |  |
| Kyushu | Kitakyushu-1 | *Hynobius stejnegeri*  *Hynobius oyamai* | 2023 | 1  8 |  |  |  |  |
| Kyushu | Kitakyushu-2 | *Hynobius nebulosus*  *Cynops pyrrhogaster* | 2023 | 4  22 |  |  |  |  |
| Kyushu | Kitakyushu-2 | *Lithobates catesbeianus* | 2023 | 1 |  |  |  |  |
| Kyushu | Kurahisa | *Cynops pyrrhogaster* | 2023 | 4 |  |  |  |  |
| Kyushu | Takeo | *Hynobius nebulosus*  *Cynops pyrrhogaster* | 2023 | 3  21 |  |  | 1 | 1 |
| Kyushu | Tensan, Karatsu | *Hynobius naevius* | 2023 | 2 |  |  |  |  |
| Kyushu | Sonobe | *Pelophylax nigromaculatus*  *Cynops pyrrhogaster* | 2023 | 1  1 |  |  |  |  |
| Kyushu | Kiyama | *Cynops pyrrhogaster* | 2023 | 15 |  |  |  |  |
| Okinawa | Onna | *Echinotriton andersoni*  *Cynops ensicauda* | 2023 | 1  3 |  |  |  |  |
| Okinawa | Henoko | *Cynops ensicauda* | 2023 | 16 |  |  |  |  |
| Okinawa | Okinawa | *Lithobates catesbeianus* | 2023 | 2 |  |  |  |  |
| Honshu | Kyoto-1 | *Andrias japonicus* | 2014 | 1 |  |  |  |  |
| Honshu | Kyoto-2 | *Andrias davidianus* | 2014 | 1 |  |  |  |  |
| Honshu | Kyoto-3 | *Andrias japonicus* | 2014 | 1 |  |  |  |  |
| Honshu | Kyoto-4 | *Andrias davidianus* | 2014 | 1 |  |  |  |  |
| Honshu | Kyoto-5 | *Andrias japonicus* | 2014 | 4 |  |  |  |  |
| Honshu | Kyoto-6 | *Andrias japonicus* | 2014 | 1 |  |  |  |  |
| Honshu | Toyooka | *Hynobius abei* | 2011 | 10 |  |  |  |  |
| Honshu | Kyotango | *Hynobius abei* | 2011 | 10 |  |  |  |  |
| Honshu | Totsugawa | *Hynobius boulengeri* | 2011 | 16 |  |  |  |  |
| Okinawa | Uruma | *Echinotriton andersoni* | 2011 | 15 |  |  |  |  |
| Honshu | Kumano | *Hynobius boulengeri* | 2011 | 1 |  |  |  |  |
| Honshu | Tsu | *Andrias japonicus* | 2011 | 1 |  |  |  |  |
| Honshu | Hinoemata | *Onychodactylus japonicus* | 2014 | 20 |  |  |  |  |
| Honshu | Sayo | *Cynops pyrrhogaster* | 2014 | 2 |  |  |  |  |
| Honshu | Shimane | *Cynops pyrrhogaster* | 2014 | 2 |  |  |  |  |
| Honshu | Kumano | *Cynops pyrrhogaster* | 2014 | 2 |  |  |  |  |
| Honshu | Toyama | *Cynops pyrrhogaster* | 2014 | 2 |  |  |  |  |
| Honshu | Toyota | *Cynops pyrrhogaster* | 2014 | 2 |  |  |  |  |
| Honshu | Awaji | *Cynops pyrrhogaster* | 2014 | 2 |  |  |  |  |
| Honshu | Seto | *Cynops pyrrhogaster* | 2014 | 2 |  |  |  |  |
| Honshu | Sekigahara | *Cynops pyrrhogaster* | 2014 | 2 |  |  |  |  |
| Honshu | Wazuka | *Cynops pyrrhogaster* | 2014 | 2 |  |  |  |  |
| Honshu | Tosashimizu | *Cynops pyrrhogaster* | 2014 | 2 |  |  |  |  |
| Honshu | Oshu | *Cynops pyrrhogaster* | 2014 | 1 |  |  |  |  |
| Honshu | Joetsu | *Cynops pyrrhogaster* | 2014 | 1 |  |  |  |  |
| Honshu | Miyoshi | *Cynops pyrrhogaster* | 2014 | 2 |  |  |  |  |
| Honshu | Kameoka | *Cynops pyrrhogaster* | 2014 | 2 |  |  |  |  |
| Honshu | Yurihonjo | *Cynops pyrrhogaster* | 2014 | 2 |  |  |  |  |
| Honshu | Futtsu | *Cynops pyrrhogaster* | 2014 | 2 |  |  |  |  |
| Honshu | Ono | *Cynops pyrrhogaster* | 2014 | 2 |  |  |  |  |
| Honshu | Sado | *Cynops pyrrhogaster* | 2014 | 2 |  |  |  |  |
| Honshu | Oshu | *Hynobius nigrescens* | 2014 | 1 |  |  |  |  |
| Honshu | Toyono | *Hynobius kimurae* | 2014 | 1 |  |  |  |  |
| Honshu | Kyoto | *Hynobius kimurae* | 2014 | 1 |  |  |  |  |
| Honshu | Ogunu | *Cynops pyrrhogaster* | 2014 | 5 |  |  |  |  |
| Kyushu | Kamiamakusa | *Cynops pyrrhogaster* | 2015 | 14 |  |  |  |  |
| Kyushu | Amakusa | *Cynops pyrrhogaster*  *Hynobius nebulosus* | 2015 | 9  3 |  |  |  |  |
| Kyushu | Oita | *Hynobius dunni* | 2015 | 14 |  |  |  |  |
| Honshu | Izu | *Onychodactylus japonicus* | 2015 | 1 |  |  |  |  |
| Honshu | Tsuchiyama | *Hynobius guttatus* | 2015 | 1 |  |  |  |  |
| Honshu | Toyama | *Cynops pyrrhogaster*  *Hynobius takedai* | 2015 | 2  7 |  |  |  |  |
| Honshu | Tanabe | *Cynops pyrrhogaster*  *Hynobius setouchi* | 2015 | 7  3 |  |  |  |  |
| Honshu | Otsu | *Hynobius kimurae* | 2015 | 1 |  |  |  |  |
| Shikoku | Mugi | *Cynops pyrrhogaster*  *Hynobius setouchi* | 2015 | 6  6 |  |  |  |  |
| Kyushu | Tano | *Hynobius dunni*  *Cynops pyrrhogaster* | 2015 | 9  3 |  |  |  |  |
| Shikoku | Kumakogen | *Cynops pyrrhogaster* | 2015 | 5 |  |  |  |  |
| Honshu | Sanda | *Cynops pyrrhogaster* | 2015 | 10 |  |  |  |  |
| Honshu | Kuki | *Cynops pyrrhogaster*  *Hynobius takedai* | 2015 | 1  1 |  |  |  |  |
| Kyushu | Nagasaki | *Cynops pyrrhogaster*  *Hynobius nebulosus* | 2015 | 5  3 |  |  | 1 |  |
| Kyushu | Hirado | *Cynops pyrrhogaster*  *Hynobius nebulosus* | 2015 | 6  5 |  |  |  |  |
| Kyushu | Iki | *Cynops pyrrhogaster* | 2015 | 2 |  |  |  |  |
| Hokkaido | Kushiro | *Salamandrella keyserlingii* | 2015 | 3 |  |  |  |  |
| Honshu | Karatsu | *Cynops pyrrhogaster* | 2015 | 6 |  |  |  |  |
| Honshu | Oshu | *Cynops pyrrhogaster*  *Hynobius nigrescens* | 2015 | 1  1 |  |  |  |  |
| Honshu | Nyu | *Cynops pyrrhogaster* | 2015 | 4 |  |  |  |  |
| Honshu | Echizen | *Cynops pyrrhogaster* | 2015 | 30 |  |  |  |  |
| Honshu | Wakasa | *Cynops pyrrhogaster* | 2015 | 8 |  |  |  |  |
| Honshu | Mikata | *Cynops pyrrhogaster* | 2015 | 12 |  |  |  |  |
| Honshu | Awara | *Cynops pyrrhogaster* | 2015 | 22 |  |  |  |  |
| Honshu | Tsuraga | *Cynops pyrrhogaster* | 2015 | 2 |  |  |  |  |
| Honshu | Ikaho | *Cynops pyrrhogaster* | 2015 | 4 |  |  |  |  |
| Honshu | Sakai | *Cynops pyrrhogaster* | 2015 | 5 |  |  |  |  |
| Honshu | Katsuyama | *Cynops pyrrhogaster* | 2015 | 5 |  |  |  |  |
| Kyushu | Kitakyushu-3 | *Cynops pyrrhogaster*  *Hynobius nebulosus* | 2023 | 3  2 |  |  |  |  |
| Kyushu | Kitakyushu-4 | *Cynops pyrrhogaster*  *Hynobius nebulosus* | 2023 | 7  9 |  |  | 2 | 1 |
| Hokkaido | Kushiro-1 | *Salamandrella keyserlingii* | 2023 | 18 |  |  |  |  |
| Hokkaido | Kushiro-2 | *Salamandrella keyserlingii* | 2023 | 11 |  |  |  |  |
| Hokkaido | Kushiro-3 | *Salamandrella keyserlingii* | 2023 | 6 |  |  |  |  |
| Hokkaido | Kushiro-4 | *Hynobius retardatus* | 2023 | 6 |  |  | 1 | 1 |
| Hokkaido | Kushiro-5 | *Hynobius retardatus* | 2023 | 2 |  |  |  |  |
| Hokkaido | Kushiro-6 | *Hynobius retardatus* | 2023 | 15 |  |  |  | 1 |
| Hokkaido | Kushiro-7 | *Salamandrella keyserlingii* | 2023 | 1 |  |  |  |  |
| Hokkaido | Kamishihoro | *Salamandrella keyserlingii* | 2023 | 2 |  |  |  |  |
| Hokkaido | Rikubetsu | *Hynobius retardatus* | 2023 | 10 |  |  |  |  |
| Honshu | Kasama | *Cynops pyrrhogaster* | 2023 | 1 |  |  |  |  |
| Honshu | Itoigawa | *Cynops pyrrhogaster* | 2023 | 1 |  |  | 1 |  |
| Honshu | Nikko | *Hynobius nigrescens* | 2023 | 4 |  |  |  |  |
| Honshu | Ichinoseki | *Cynops pyrrhogaster* | 2023 | 3 |  |  |  |  |
| Honshu | Tsukazawa | *Cynops pyrrhogaster* | 2023 | 5 |  |  |  |  |
| Honshu | Sakuragawa | *Onychodactylus tsukubaensis* | 2023 | 2 |  |  |  |  |
| Honshu | Hinoemata | *Onychodactylus japonicus* | 2023 | 4 |  |  |  |  |
