# Supplementary material for: Lack of variations in the salamander chytrid fungus, Batrachochytrium salamandrivorans, at its alleged origin: Updating its Japanese distribution with new evidence
Source: PLoS One. 2024 Jun 13;19(6):e0305228. doi: 10.1371/journal.pone.0305228 (PMC11175521; doi:10.1371/journal.pone.0305228)
Supplement: Attachment — Submitted filename: Review_Lastra_et_al_PLoS_ONE.docx [file pone.0305228.s002.docx]

This is an interesting study in the field of Bsal research, which is a dangerous amphibian pathogen in certain parts of the world, so far especially Europe. As Asia is considered the origin of Bsal, this study is an important contribution bringing to light novel knowledge. The “story” is sound, the introduction leads to the purpose of the paper and the methods are standard methods. Results are well described and convincing. The discussion refers to the significant points and emphasizes the importance of the finding in a critical view.

I like the paper and will be happy to see it published in PLoS ONE. I made some comments, see below, which might help the authors to improve their paper.

However, before acceptance, in many parts, the language is awkward. Maybe look for a native speaker to make corrections.

**Title**, line 1: I find the title a bit tough, as Bsal is not always a killer, especially not in Japan. I recommend to change the title to simply use the term “salamander chytrid fungus”

**Introduction**, line 53: Maybe rewrite as “in many parts of the world” instead of worldwide.

Line 59-63: True but a bit out of focus of the paper, I suggest to be more brief here.

Line 66 f: Emphasize here that this study and others (e.g. 6, 11, 12) have suggested already that Bsal is of Asian origin.

Line 84f. You should state here that also Bd was in your focus, as you studied both (mentioned in line 109).

**Methods**, line 94: delete “different” – 22 species, by definition, are different.

**Results**, line 128f.: Although important this I part of the method and was mentioned there already (line 114f.). Do not repeat method here. Move details to method section and rewrite.

Line 145f.: Add the reference number to the citation of Bloii et al. and Spitzen-van der Sluijs et al.

**Discussion**, line 164: “This evidence suggests a long-term relationship among host and pathogen…” Explain this a bit better.

Line 171f: “Bsal qPCR protocol was under discussion and considered not enough to claim a sample for being Bsal positive [17]. Thus, in summary. the qPCR Bsal protocol was said to tend to create false positives.” This likewise needs a better explanation for the reader.

Line 177: Yes, I agree you followed a “conservative” approach but still samples were not studied in different labs, which sometimes is considered a critical aspect, as well. Please consider to include this into the discussion here.

Line 193: Say “suggested sister species”.
