# Supplementary material for: Lack of variations in the salamander chytrid fungus, Batrachochytrium salamandrivorans, at its alleged origin: Updating its Japanese distribution with new evidence
Source: PLoS One. 2024 Jun 13;19(6):e0305228. doi: 10.1371/journal.pone.0305228 (PMC11175521; doi:10.1371/journal.pone.0305228)
Supplement: Attachment — Submitted filename: 2-Response_to_Reviewers.docx [file pone.0305228.s003.docx]

**Response to Reviewers to PONE-D-23-38081**

**Disentangling the origin of the salamander killer fungus, *Batrachochytrium salamandrivorans*: Updating its Japanese distribution with new evidence**

Journal requirements:

1. When submitting your revision, we need you to address these additional requirements.

Please ensure that your manuscript meets PLOS ONE's style requirements, including those for file naming. The PLOS ONE style templates can be found at 
[https://journals.plos.org/plosone/s/file?id=wjVg/PLOSOne_formatting_sample_main_body.pdf](https://mcas-proxyweb.mcas.ms/certificate-checker?login=false&originalUrl=https%3A%2F%2Fjournals.plos.org.mcas.ms%2Fplosone%2Fs%2Ffile%3Fid%3DwjVg%2FPLOSOne_formatting_sample_main_body.pdf%26McasTsid%3D20893&McasCSRF=3c31b6fb70006f3652fa6b22592e882554601869fd83588c0d7bc5da3adf1f4d) and 
[https://journals.plos.org/plosone/s/file?id=ba62/PLOSOne_formatting_sample_title_authors_affiliations.pdf](https://mcas-proxyweb.mcas.ms/certificate-checker?login=false&originalUrl=https%3A%2F%2Fjournals.plos.org.mcas.ms%2Fplosone%2Fs%2Ffile%3Fid%3Dba62%2FPLOSOne_formatting_sample_title_authors_affiliations.pdf%26McasTsid%3D20893&McasCSRF=3c31b6fb70006f3652fa6b22592e882554601869fd83588c0d7bc5da3adf1f4d).

*-DLG: Revised and modified.*

2. We note that the grant information you provided in the ‘Funding Information’ and ‘Financial Disclosure’ sections do not match.

When you resubmit, please ensure that you provide the correct grant numbers for the awards you received for your study in the ‘Funding Information’ section.

*-DLG: Revised and modified.*

3. Thank you for stating the following financial disclosure: 
 [David Lastra González is supported by the JSPS Postdoctoral fellowships for Research in Japan].  
Please state what role the funders took in the study.  If the funders had no role, please state: ""The funders had no role in study design, data collection and analysis, decision to publish, or preparation of the manuscript."" 
If this statement is not correct you must amend it as needed. 
Please include this amended Role of Funder statement in your cover letter; we will change the online submission form on your behalf.

*- DLG: Done*

4. Ethics statement appears in the Methods section of the manuscript AND at the end of the manuscript:
Your ethics statement should only appear in the Methods section of your manuscript. If your ethics statement is written in any section besides the Methods, please delete it from any other section.

*- DLG: Solved*

5. We note that Figure 1 in your submission contain [map/satellite] images which may be copyrighted. All PLOS content is published under the Creative Commons Attribution License (CC BY 4.0), which means that the manuscript, images, and Supporting Information files will be freely available online, and any third party is permitted to access, download, copy, distribute, and use these materials in any way, even commercially, with proper attribution. For these reasons, we cannot publish previously copyrighted maps or satellite images created using proprietary data, such as Google software (Google Maps, Street View, and Earth). For more information, see our copyright guidelines: [http://journals.plos.org/plosone/s/licenses-and-copyright](https://mcas-proxyweb.mcas.ms/certificate-checker?login=false&originalUrl=http%3A%2F%2Fjournals.plos.org.mcas.ms%2Fplosone%2Fs%2Flicenses-and-copyright%3FMcasTsid%3D20893&McasCSRF=3c31b6fb70006f3652fa6b22592e882554601869fd83588c0d7bc5da3adf1f4d).
 
We require you to either (1) present written permission from the copyright holder to publish these figures specifically under the CC BY 4.0 license, or (2) remove the figures from your submission:
 
a. You may seek permission from the original copyright holder of Figure 1 to publish the content specifically under the CC BY 4.0 license.  
 
We recommend that you contact the original copyright holder with the Content Permission Form ([http://journals.plos.org/plosone/s/file?id=7c09/content-permission-form.pdf](https://mcas-proxyweb.mcas.ms/certificate-checker?login=false&originalUrl=http%3A%2F%2Fjournals.plos.org.mcas.ms%2Fplosone%2Fs%2Ffile%3Fid%3D7c09%2Fcontent-permission-form.pdf%26McasTsid%3D20893&McasCSRF=3c31b6fb70006f3652fa6b22592e882554601869fd83588c0d7bc5da3adf1f4d)) and the following text:
“I request permission for the open-access journal PLOS ONE to publish XXX under the Creative Commons Attribution License (CCAL) CC BY 4.0 ([http://creativecommons.org/licenses/by/4.0/](https://mcas-proxyweb.mcas.ms/certificate-checker?login=false&originalUrl=http%3A%2F%2Fcreativecommons.org.mcas.ms%2Flicenses%2Fby%2F4.0%2F%3FMcasTsid%3D20893&McasCSRF=3c31b6fb70006f3652fa6b22592e882554601869fd83588c0d7bc5da3adf1f4d)). Please be aware that this license allows unrestricted use and distribution, even commercially, by third parties. Please reply and provide explicit written permission to publish XXX under a CC BY license and complete the attached form.”
 
Please upload the completed Content Permission Form or other proof of granted permissions as an ""Other"" file with your submission.
 
In the figure caption of the copyrighted figure, please include the following text: “Reprinted from [ref] under a CC BY license, with permission from [name of publisher], original copyright [original copyright year].”

- *DLG: Revised and modified.*

b. If you are unable to obtain permission from the original copyright holder to publish these figures under the CC BY 4.0 license or if the copyright holder’s requirements are incompatible with the CC BY 4.0 license, please either i) remove the figure or ii) supply a replacement figure that complies with the CC BY 4.0 license. Please check copyright information on all replacement figures and update the figure caption with source information. If applicable, please specify in the figure caption text when a figure is similar but not identical to the original image and is therefore for illustrative purposes only.
The following resources for replacing copyrighted map figures may be helpful:
 
USGS National Map Viewer (public domain): [http://viewer.nationalmap.gov/viewer/](https://mcas-proxyweb.mcas.ms/certificate-checker?login=false&originalUrl=http%3A%2F%2Fviewer.nationalmap.gov.mcas.ms%2Fviewer%2F%3FMcasTsid%3D20893&McasCSRF=3c31b6fb70006f3652fa6b22592e882554601869fd83588c0d7bc5da3adf1f4d)
The Gateway to Astronaut Photography of Earth (public domain): [http://eol.jsc.nasa.gov/sseop/clickmap/](https://mcas-proxyweb.mcas.ms/certificate-checker?login=false&originalUrl=http%3A%2F%2Feol.jsc.nasa.gov.mcas.ms%2Fsseop%2Fclickmap%2F%3FMcasTsid%3D20893&McasCSRF=3c31b6fb70006f3652fa6b22592e882554601869fd83588c0d7bc5da3adf1f4d)
Maps at the CIA (public domain): [https://www.cia.gov/library/publications/the-world-factbook/index.html](https://mcas-proxyweb.mcas.ms/certificate-checker?login=false&originalUrl=https%3A%2F%2Fwww.cia.gov.mcas.ms%2Flibrary%2Fpublications%2Fthe-world-factbook%2Findex.html%3FMcasTsid%3D20893&McasCSRF=3c31b6fb70006f3652fa6b22592e882554601869fd83588c0d7bc5da3adf1f4d) and [https://www.cia.gov/library/publications/cia-maps-publications/index.html](https://mcas-proxyweb.mcas.ms/certificate-checker?login=false&originalUrl=https%3A%2F%2Fwww.cia.gov.mcas.ms%2Flibrary%2Fpublications%2Fcia-maps-publications%2Findex.html%3FMcasTsid%3D20893&McasCSRF=3c31b6fb70006f3652fa6b22592e882554601869fd83588c0d7bc5da3adf1f4d)
NASA Earth Observatory (public domain): [http://earthobservatory.nasa.gov/](https://mcas-proxyweb.mcas.ms/certificate-checker?login=false&originalUrl=http%3A%2F%2Fearthobservatory.nasa.gov.mcas.ms%2F%3FMcasTsid%3D20893&McasCSRF=3c31b6fb70006f3652fa6b22592e882554601869fd83588c0d7bc5da3adf1f4d)
Landsat: [http://landsat.visibleearth.nasa.gov/](https://mcas-proxyweb.mcas.ms/certificate-checker?login=false&originalUrl=http%3A%2F%2Flandsat.visibleearth.nasa.gov.mcas.ms%2F%3FMcasTsid%3D20893&McasCSRF=3c31b6fb70006f3652fa6b22592e882554601869fd83588c0d7bc5da3adf1f4d)
USGS EROS (Earth Resources Observatory and Science (EROS) Center) (public domain): [http://eros.usgs.gov/#](https://mcas-proxyweb.mcas.ms/certificate-checker?login=false&originalUrl=http%3A%2F%2Feros.usgs.gov.mcas.ms%2F%3FMcasTsid%3D20893&McasCSRF=3c31b6fb70006f3652fa6b22592e882554601869fd83588c0d7bc5da3adf1f4d)
Natural Earth (public domain): [http://www.naturalearthdata.com/](https://mcas-proxyweb.mcas.ms/certificate-checker?login=false&originalUrl=http%3A%2F%2Fwww.naturalearthdata.com.mcas.ms%2F%3FMcasTsid%3D20893&McasCSRF=3c31b6fb70006f3652fa6b22592e882554601869fd83588c0d7bc5da3adf1f4d)

6. Please include captions for your Supporting Information files at the end of your manuscript, and update any in-text citations to match accordingly. Please see our Supporting Information guidelines for more information: [http://journals.plos.org/plosone/s/supporting-information](https://mcas-proxyweb.mcas.ms/certificate-checker?login=false&originalUrl=http%3A%2F%2Fjournals.plos.org.mcas.ms%2Fplosone%2Fs%2Fsupporting-information%3FMcasTsid%3D20893&McasCSRF=3c31b6fb70006f3652fa6b22592e882554601869fd83588c0d7bc5da3adf1f4d).

*- DLG: Solved*

[Note: HTML markup is below. Please do not edit.]

Reviewers' comments:
Reviewer's Responses to Questions

**Comments to the Author**

1. Is the manuscript technically sound, and do the data support the conclusions?

The manuscript must describe a technically sound piece of scientific research with data that supports the conclusions. Experiments must have been conducted rigorously, with appropriate controls, replication, and sample sizes. The conclusions must be drawn appropriately based on the data presented.

Reviewer #1: Yes

Reviewer #2: Partly

2. Has the statistical analysis been performed appropriately and rigorously?

Reviewer #1: Yes

Reviewer #2: N/A

3. Have the authors made all data underlying the findings in their manuscript fully available?

The [PLOS Data policy](https://mcas-proxyweb.mcas.ms/certificate-checker?login=false&originalUrl=http%3A%2F%2Fwww.plosone.org.mcas.ms%2Fstatic%2Fpolicies.action%3FMcasTsid%3D20893%23sharing&McasCSRF=3c31b6fb70006f3652fa6b22592e882554601869fd83588c0d7bc5da3adf1f4d) requires authors to make all data underlying the findings described in their manuscript fully available without restriction, with rare exception (please refer to the Data Availability Statement in the manuscript PDF file). The data should be provided as part of the manuscript or its supporting information, or deposited to a public repository. For example, in addition to summary statistics, the data points behind means, medians and variance measures should be available. If there are restrictions on publicly sharing data—e.g. participant privacy or use of data from a third party—those must be specified.

Reviewer #1: Yes

Reviewer #2: Yes

4. Is the manuscript presented in an intelligible fashion and written in standard English?

PLOS ONE does not copyedit accepted manuscripts, so the language in submitted articles must be clear, correct, and unambiguous. Any typographical or grammatical errors should be corrected at revision, so please note any specific errors here.

Reviewer #1: No

Reviewer #2: No

5. Review Comments to the Author

Please use the space provided to explain your answers to the questions above. You may also include additional comments for the author, including concerns about dual publication, research ethics, or publication ethics. (Please upload your review as an attachment if it exceeds 20,000 characters)

Reviewer #1: This is an interesting study in the field of Bsal research, which is a dangerous amphibian pathogen in certain parts of the world, so far especially Europe. As Asia is considered the origin of Bsal, this study is an important contribution bringing to light novel knowledge. The “story” is sound, the introduction leads to the purpose of the paper and the methods are standard methods. Results are well described and convincing. The discussion refers to the significant points and emphasizes the importance of the finding in a critical view.
I like the paper and will be happy to see it published in PLoS ONE. I made some comments, see below, which might help the authors to improve their paper.
However, before acceptance, in many parts, the language is awkward. Maybe look for a native speaker to make corrections.

*- DLG: Corrections according to your comments have been included, I appreciate your time for improving this manuscript. Native speaker has also checked the manuscript. Kind regards.*

Title, line 1: I find the title a bit tough, as Bsal is not always a killer, especially not in Japan. I recommend to change the title to simply use the term “salamander chytrid fungus”

*- DLG: Solved*

Introduction, line 53: Maybe rewrite as “in many parts of the world” instead of worldwide.

*- DLG: Solved*

Line 59-63: True but a bit out of focus of the paper, I suggest to be more brief here.

*- DLG: Done*

Line 66: Emphasize here that this study and others (e.g. 6, 11, 12) have suggested already that Bsal is of Asian origin.

*- DLG: Done*

Line 84f. You should state here that also Bd was in your focus, as you studied both (mentioned in line 109).

*- DLG: Done*

Methods, line 94: delete “different” – 22 species, by definition, are different.

*- DLG: Done*

Results, line 128f.: Although important this I part of the method and was mentioned there already (line 114f.). Do not repeat method here. Move details to method section and rewrite.

*- DLG: Done*

Line 145f.: Add the reference number to the citation of Bloii et al. and Spitzen-van der Sluijs et al.

*- DLG: Done. Just adding the reference number,as suggested, but I have not deleted the author’s name to keep the table as self-explained.*

Discussion, line 164: “This evidence suggests a long-term relationship among host and pathogen…” Explain this a bit better.

*- DLG: Done in text as well: The confirmed presence of the pathogen for more than a decade, the lack of individuals with high infection loads or detected outbreaks do not show the same interactions of a novel pathogen into its potential hosts as it happened in Central Europe*

Line 171f: “Bsal qPCR protocol was under discussion and considered not enough to claim a sample for being Bsal positive [17]. Thus, in summary. the qPCR Bsal protocol was said to tend to create false positives.” This likewise needs a better explanation for the reader.

*- DLG: Not sure how to do that briefly, as it is an important topic of discussion that would require a substantial space and it is not within the scope of the article, neither a personal claim. I hope that the reference of Thomas et al. 2018 will clarify to the reader this topic. Even my personal opinion, (and not necessarily the opinion of all my co-authors) does not entirely align with this affirmation, because it depends on many factors which the referenced article did not entirely take into account. As I mentioned, it is a highly complex topic, beyond the discussion of this article. Nevertheless, I will be more than happy to have a discussion about it with the reviewer.*

Line 177: Yes, I agree you followed a “conservative” approach but still samples were not studied in different labs, which sometimes is considered a critical aspect, as well. Please consider to include this into the discussion here.

*- DLG: Done in text as well: On the other hand, we could have followed even a more conservative approach if we had confirmed the Bsal positive samples in two laboratories.*

Line 193: Say “suggested sister species”.

*- DLG: Done. I am curious about if the reviewer considers not proven or have doubts about this matter. Do you think that Bd could not be the sister species of Bsal or it is more a technicality of the sister species expression?*

Reviewer #2: Summary
This paper presents data on the prevalence of the fungal pathogen Bsal (and its congener Bd) in samples collected from multiple Japanese islands (as well as 2 samples from Myanmar). The authors find a low prevalence of Bsal-positive samples (comparable to that in other surveys in East and Southeast Asia), but some inconsistencies in detection between different diagnostic methods. In my opinion, the most useful contributions from this paper are the establishment of new Bsal localities in East Asia, some associated sequence data, and the detection in a species with no previous record of Bsal.
Major concerns
The paper is in need of substantial English-language editing throughout. The meaning of the text is often clear, but there are key passages where it is not, and key terms are not clearly defined. For example, it is hard to tell what species is being referred to in the description of the outbreak in NE Spain (Line 57)

*- DLG: Solved. I think that I could detect what you mentioned rightly. I think that an incomplete sentence was the cause of being hard to read, more than a language problem.*

There are a few places where I think the conclusions and interpretations don’t seem to be in line with the data. For example, the suggestion that “this part of Asia is the chytrid hotspot” in the abstract doesn’t seem consistent with their results and interpretations, but it is hard to tell if this is a language issue or an interpretation problem.

*- DLG: Not a language issue I think. The combination of many Bd haplotypes together with the detection of Bsal in several independent spots made that the authors think that Okinawa could be considered as a hotspot for chytrids.*

Also, I think the authors should be more cautious about interpretations related to long-term endemism based on the limited data they provide.

*- DLG: My apologies if I could not transmit the caution that we have used for writing this manuscript. I have modified that sentence to be less assertive.*

The organization of the Introduction could be much clearer, including both the flow of ideas within paragraphs and the relationships between paragraphs. For example, there is a rather abrupt transition from Bsal to Bd on line 74, and there is a series of very short paragraphs between lines 59 and 71 that seem too small to stand on their own and don’t really flow together very well.

*- DLG: This abrupt transition is intended to advise the reader to not focus only on Bsal, but in the area of study as well. Having a background information about chytridiomycosis in Okinawa (Ryukyu Islands) was considered important.*

It would be good to have more discussion of why they got disparate results using different detection methods.

*- DLG: As the reviewer noticed, this is an interesting fact, and we are working in an independent article to fully explain this circumstance. We are glad that you consider it an intriguing situation as well.*

Specific comments
Line 54: Are the lesions themselves lethal? Or are they a symptom associated with severe disease, which causes death through other mechanisms?

*- DLG: Yes, they could be lethal, but at the same times they are the result of highly advanced stages of the disease. In the case of Bsal, unlike Bd that does not provoke ulcera, the ulcera can be a lethal lesion. Although, in the wild is hard to see these stages and the typical cause of death is an impairment of the ability to keep homeostasis*.

Lines 56-58: Rewrite sentence for clarity.

*- DLG: Done: It has caused a severe decline in Belgium and Germany of the fire salamander as well as an outbreak in Triturus marmoratus in NE Spain due to an illegal release of allochthonous species that affected local species as well*

Lines 59-63: Good points, but are not very clearly connected to the surrounding material in the Introduction, and may be less relevant for this particular study.

*- DLG: Done. I have reduced them. Being the first paper focusing only on Bsal in Japan I thought that could be important to summarize this information about what has done in Europe to promote similar actions here.*

Lines 80-82: Meaning not clear

*- DLG: I have added a word that was missing. In these sentences, we were trying to explain that in contrast to the previous paragraph (where we explained that in Japan, Bd has many haplotypes) the Bsal amplicons that we found belong to the known Bsal isolate. In summary, for the same area Bd has many haplotypes and for Bsal the same haplotype.*

Line 95: The Introduction focuses on Japan, so I was a little bit surprised to see that there were samples from Myanmar as well. I didn’t see these mentioned anywhere else in the paper.

*- DLG: Yes, you are right. Fortunately, these two samples do not have any relevant information regarding chytrids. They were available thanks to a collaborator, so they had to be included in the dataset, but there is nothing much to say more than the information that is provided in the Supplementary information.*

Line 117: Unclear what the 2nd derivative maximum was used for.

*- DLG: The 2^nd^ derivative maximum is the standard procedure that it is followed to analyse chytrid samples by quantitative PCR. Basically, from the different options that the software gives during the analysis, the selected one was the 2^nd^ derivative maximum. The Lightcycler 480 software belongs to the Roche Lightcycler 480 II quantitative PCR.*

Table 1: Would be good to have the total number of samples per island listed somewhere

*- DLG: That information is available within the Supplementary Information of this manuscript, where you can find detailed information about the origin of each sample.*

Line 162: I’m not sure why sampling effort would be related to infection load

*- DLG: You are completely right that how it is stated right know it could induce that both things are related. I meant that, even though we have a large dataset we did not detect any animal with signs of a severe infection. Anyway, I have modified this part in the text for a better understanding.*

Lines 164-165: Not clear how the data suggests long-term relationship and endemism.

*- DLG: I have added a longer explanation in the text, as follows: The confirmed presence of the pathogen for more than a decade, the lack of individuals with high infection loads, and a low prevalence without detected outbreaks respond better to an endemic situation in a relationship between host and pathogen. If we keep in mind the situation and how was the arrival of Bsal into the European amphibian populations, it does not show the same interactions of a novel pathogen into its potential hosts as it happened in Central Europe.*

Lines 170-180: The discussion of different diagnostic techniques seems useful. Would be good to flesh this out more, including adding more information about the differences in sensitivity between the assays used here and other approaches.

*- DLG: Yes, it looks that there are differences in sensitivity. Unfortunately, we cannot provide yet these values, we are working in an independent article to fully explain this circumstance.*

Line 186-188: Do we know that harsh winters decrease probability of detecting Bsal? Presumably the hosts are in habitats that are not quite so cold?

*- DLG: Yes, and it depends. Yes, harsh winters would decrease the activity of Bsal or even could kill it. To date, the temperature range for Bsal activity is between 4º-25º C with an optimal around 15ºC. And it depends because our area of study was Japan, where in the north (Hokkaido) the temperatures can be very extreme (average temperature ranges from −12 to −4 °C in January) or in the south (Okinawa) there is no winter (minimum temperature is around 13ºC).*

Lines 191-192: I agree that it is interesting and useful to expand the list of species on which Bsal has been detected.
Line 191-205: nice paragraph

*- DLG: Thank you for appreciating that.*

Lines 210-211: I don’t think the current paper has enough data to support this point, and I’m not sure I agree with the logic (eg, there can be species in the introduced range that also tend to have low Bsal loads).

*- DLG: Yes, I agree with you, and I was doubting if to include deeper and more varied options. I will do it then. In the text are as follows: However, similar relationships have been already found in Europe (e.g. Ichtyosaura alpestris [22] even though Bsal does not show any sign of being native to Europe. Strong statements should be avoided with the current known information.*

Lines 214-216: This sentence doesn’t seem to fit very well

*- DLG: Modified to have a broader scenario and show the complexity of the Bsal origin and its distribution.*

Map figure: would be useful if this showed all sampled localities (not just the Bsal-positive ones)

*- DLG: That was the initial plan, but due to the number of localities, Japan’s shape and the difference in size among islands, the result was quite ugly and much information was missing due to the overlapping of localities, especially in the smaller islands. To give that information but in a not graphical way, we included the supplementary information.*

6. PLOS authors have the option to publish the peer review history of their article ([what does this mean?](https://mcas-proxyweb.mcas.ms/certificate-checker?login=false&originalUrl=https%3A%2F%2Fjournals.plos.org.mcas.ms%2Fplosone%2Fs%2Feditorial-and-peer-review-process%3FMcasTsid%3D20893%23loc-peer-review-history&McasCSRF=3c31b6fb70006f3652fa6b22592e882554601869fd83588c0d7bc5da3adf1f4d)). If published, this will include your full peer review and any attached files.


If you choose “no”, your identity will remain anonymous but your review may still be made public.


**Do you want your identity to be public for this peer review?** For information about this choice, including consent withdrawal, please see our [Privacy Policy](https://mcas-proxyweb.mcas.ms/certificate-checker?login=false&originalUrl=https%3A%2F%2Fwww.plos.org.mcas.ms%2Fprivacy-policy%3FMcasTsid%3D20893&McasCSRF=3c31b6fb70006f3652fa6b22592e882554601869fd83588c0d7bc5da3adf1f4d).

Reviewer #1: No

Reviewer #2: No

[NOTE: If reviewer comments were submitted as an attachment file, they will be attached to this email and accessible via the submission site. Please log into your account, locate the manuscript record, and check for the action link "View Attachments". If this link does not appear, there are no attachment files.]

While revising your submission, please upload your figure files to the Preflight Analysis and Conversion Engine (PACE) digital diagnostic tool, [https://pacev2.apexcovantage.com/](https://mcas-proxyweb.mcas.ms/certificate-checker?login=false&originalUrl=https%3A%2F%2Fpacev2.apexcovantage.com.mcas.ms%2F%3FMcasTsid%3D20893&McasCSRF=3c31b6fb70006f3652fa6b22592e882554601869fd83588c0d7bc5da3adf1f4d). PACE helps ensure that figures meet PLOS requirements. To use PACE, you must first register as a user. Registration is free. Then, login and navigate to the UPLOAD tab, where you will find detailed instructions on how to use the tool. If you encounter any issues or have any questions when using PACE, please email PLOS at [figures@plos.org](mailto:figures@plos.org). Please note that Supporting Information files do not need this step.
