# Supplementary material for: Lack of variations in the salamander chytrid fungus, Batrachochytrium salamandrivorans, at its alleged origin: Updating its Japanese distribution with new evidence
Source: PLoS One. 2024 Jun 13;19(6):e0305228. doi: 10.1371/journal.pone.0305228 (PMC11175521; doi:10.1371/journal.pone.0305228)
Supplement: Attachment — Submitted filename: 2-Response_to_reviewers.docx [file pone.0305228.s004.docx]

Reviewers' comments:

Reviewer's Responses to Questions

**Comments to the Author**

6. Review Comments to the Author

Please use the space provided to explain your answers to the questions above. You may also include additional comments for the author, including concerns about dual publication, research ethics, or publication ethics. (Please upload your review as an attachment if it exceeds 20,000 characters)

Reviewer #1: (No Response)

Reviewer #3: The authors describe an extensive sampling of amphibians throughout Japan for chytrid fungi. Results are largely confirmatory of previous studies (eg Goka et al., 2009; Martel et al., 2014) but are sufficiently interesting to warrant publication. While in general, the study is fine, I would recommend addressing several issues:

Major concerns:
I only have one major concern and that is that sequencing a short (how much bp? Please mention) part of the ITS of the chytrid fungus B. salamandrivorans is not sufficient to make any inference about diversity. I am not sure whether I understood correctly, but sequences would all be 100% identical (and identical to the available sequence). The authors really should refrain from drawing any conclusions with regard to diversity / phylogeny based on these results. At most, the sequencing confirms that a short B. salamandrivorans ITS sequence is present in their samples. Hence, conclusions with regard to “origin”, “hotspot” or any inference with regard to epidemiology (eg recent colonization) must not be drawn. Such statements should be omitted from title, abstract and discussion, unless the authors would have performed additional analyses that do warrant conclusions with regard to B. salamandrivorans strain typing (eg strain isolation and sequencing).

- *DLG: The obtained sequences are 160 base pairs long, it has been included in the text. I understand your claim about the use of the terms “origin” and “hotspot”. About the term “origin”, we are not claiming to know the origin of Bsal, we are only adding data to the current knowledge with the aim of contributing to answer the question about the origin of Bsal. And that is why we have used the expression of disentangling, trying to not claim to solve or to know the origin of Bsal. In the case of the term “hotspot”, we are not focusing our discussion exclusively on Bsal. We meant to say that Okinawa is a hotspot for chytrids because of the combined presence of Bd and Bsal. On the other hand, we have no special interest of this term, and it has been modified according to the reviewer’s suggestion. And lastly, about the recent colonization, we have included modifications in the text to solve these concerns following your recommendations “*Nonetheless, we are aware that the sequencing of a short fragment from the ITS region falls short for a firm interpretation, and additional methodologies such as Bsal strain typing would be recommended to infer sound conclusions.*” (line 216).*

Minor concerns:

1) There is an odd mentioning of two samples from Myanmar. It is difficult to understand how this fits the story. Suggestion to omit from this paper (does not really add anything useful). Also, if the authors would prefer to leave these samples in, please provide all permits (incl Nagoya protocol) from Myanmar to demonstrate legal original (+ correct the name in S1 to Tylototriton). However, there does not appear to be any sound, scientific rationale to include these two samples.

*-DLG: After discussing it with the co-authors, the samples from Myanmar have been removed from the manuscript.*

2) Results need revision; the table 1 is confusing and at the same time does not provide sufficient information. For example, the difference between the second and third column is unclear. The total number of sampled localities per island + the number of samples (pooled or not) examined should be summarized here. The same for the legend of S Table, which is equally difficult to understand. E.g. the legend states “detected by Blooi et al…”, where I guess the authors mean “detected by qPCR (according to Blooi et al.).

*-DLG: Corrections have been included according to the reviewer’s comments.*

3) It is difficult to track any data that refer to prevalence or infection load. The authors used pooled and simple samples. This is ok, but pooling samples requires any conclusion to prevalence and infection loads to be nuanced (eg prevalence then is at pool level).

*-DLG: The pooling of the samples did not affect to the prevalence levels or infection loads as the pooling did not affect to the Okinawan samples nor other localities with Bsal positives.*

4) There is one issue where I would love to see an additional sentence and that is the potential of false positives (which includes the possibility of another organism with the same (short) ITS sequence). According to WOAH, diagnosis of B. salamandrivorans infection requires the use of two independent techniques (eg culturing or histopathology). Sequencing amplicons from a positive PCR is not considered an independent technique. I would like to stress this is not meant to disregard the authors’ results. I fully realize it is very difficult, if not impossible, to use an additional technique (culturing, histopathology), especially in low level infected populations. However, if not performed, it is impossible to discard the possibility of false positives (which has been the case in Europe, see eg Thomas et al. and which remains unexplained). I would therefore recommend to add a sentence along these lines: “Although qPCR is an established, reliable and robust technique and amplicons were identical to the published B. salamandrivorans sequence, the low level infections in the Japanese amphibians preclude the use of an independent diagnostic technique as required by the WOAH, hampering the possibility to exclude false positive results.”

*-DLG: The sentence has been included as suggested by the reviewer.*
