# Supplementary material for: Lack of variations in the salamander chytrid fungus, Batrachochytrium salamandrivorans, at its alleged origin: Updating its Japanese distribution with new evidence
Source: PLoS One. 2024 Jun 13;19(6):e0305228. doi: 10.1371/journal.pone.0305228 (PMC11175521; doi:10.1371/journal.pone.0305228)
Supplement: Attachment — Submitted filename: 2-Response_to_reviewers.docx [file pone.0305228.s005.docx]

Reviewers' comments:

Reviewer's Responses to Questions

**Comments to the Author**

6. Review Comments to the Author

Please use the space provided to explain your answers to the questions above. You may also include additional comments for the author, including concerns about dual publication, research ethics, or publication ethics. (Please upload your review as an attachment if it exceeds 20,000 characters)

Reviewer #1: (No Response)

Reviewer #3: Concerns have been addressed sufficiently. I commend the authors for their extensive study and have no further concerns.

Reviewer #4: The title is inaccurate. Their is no phylogenetic analysis in this paper or comparative measures of genetic diversity. A more appropriate title could be: 'An updated distribution of Batrachochytrium salamandrivorans in Japan’.

- *DLG: By your suggestion to modify the title, I understand that you claim to be inaccurate because of the “Disentangling the origin…” part of the title. About the term “origin”, we are not claiming to know the origin of Bsal, we are only adding data to the current knowledge with the aim of contributing to answer the question about the origin of Bsal. And that is why we have used the expression of disentangling, trying to not claim to solve or to know the origin of Bsal.*

  L41. It's prevalence - not incidence. And it is not high - it is actually very low compared to outbreak areas in Europe.
- *DLG: The use of these terms often provokes misunderstandings. In our case, we have used the word incidence following the same meaning as Noordzij et al. 2010. In other words, the prevalence reflects the number of existing cases of a disease. In contrast, the incidence reflects the number of* ***new*** *cases of disease and can be reported as a risk, as in the article Martel et al. 2013 did for the new different cases of Bsal. Therefore, you are right, if we would have meant prevalence, it is low prevalence comparing the European outbreaks.*

L48. 'Low variability' is a relative term. What are you comparing against? Epidemiologically speaking, low diversity indicates an invasion event (limited temporal time allowed for genetic diversity to accrue). So your data more accurately represents a recent invasion.

- *DLG: Yes, absolutely. We agree that epidemiologically speaking, low diversity indicates an invasion event. That is our main point of the article. In comparison of the high variability of its sister species, Bd, in Okinawa (which would mean rather the opposite and a long-term interaction between Bd and Okinawan populations of Cynops ensicauda), Bsal has low variability and as you also suggested, could be explained by a recent invasion event. This circumstance is what we find intriguing, a theoretically different time colonization between Bd and Bsal for Okinawa Island, despite of same response (susceptible) of Cynops ensicauda to both chytrids.*

L108. What is the evidence that the metabarcoding marker that you have chosen has any phylogenetic resolution? O'Hanlon et al. has shown very clearly (see Supp Info tanglegram plots) that ITS-2 has no phylogenetic resolution for Bd.

- *DLG: Nowadays, I would say that this is the main problem for Bsal, there is very little information about the Bsal phylogeny. Unfortunately, there are not many options to be chosen, so we are just following current Bsal methodology. As you pointed out fairly, much more information is known for Bd, but there is not such a kind strong evidence for Bsal and the phylogenetic resolution that current sequencing provides.*

  L131. Sanger sequencing of what locus?
- *DLG: The STerF and SterR primers were used to detect the 5.8S rRNA gene of Bsal (GenBank accession number KC762295) following the description of Blooi et al. 2013. Included in the text in the methods section.*

  L223. This is a hypothesis. For instance in Europe only Salamandra is highly susceptible. Other species of newt tolerate infection or are resistant to infection.
- *DLG: I am not entirely sure about what do you mean with this comment as current line numbers does not fit with a similar topic. So please, allow me to guess it according to your matter of interest. I think that, in the current version, your point is discussed in the line 232. In these sentences, our intention is just to mention the different scenarios that could explain the low loads detected. Of course, and we tried to highlight the hypothetical meaning of these sentences, we show to the reader how particular is the situation found in Okinawa and the relationship between Bsal and the genus Cynops, which is considered neither tolerant nor resistant according to Martel et al. 2014.*
