# Supplementary material for: Lack of variations in the salamander chytrid fungus, Batrachochytrium salamandrivorans, at its alleged origin: Updating its Japanese distribution with new evidence
Source: PLoS One. 2024 Jun 13;19(6):e0305228. doi: 10.1371/journal.pone.0305228 (PMC11175521; doi:10.1371/journal.pone.0305228)
Supplement: Attachment — Submitted filename: 2-Response_to_reviewers4.docx [file pone.0305228.s006.docx]

Reviewers' comments:

Reviewer's Responses to Questions

**Comments to the Author**

6. Review Comments to the Author

Please use the space provided to explain your answers to the questions above. You may also include additional comments for the author, including concerns about dual publication, research ethics, or publication ethics. (Please upload your review as an attachment if it exceeds 20,000 characters).

- Reviewer #1: (No Response)
- Reviewer #4: I still think the title is inaccurate. Even if your aim is to contribute to the elucidation of the origin of Bsal, your data do not say anything about this, so to include ‘“Disentangling the origin” is confusing.
- *DLG: The title has been modified as you suggested: Lack of variations in the salamander chytrid fungus, Batrachochytrium salamandrivorans, at its alleged origin: Updating its Japanese distribution with new evidence.*
- As you argue in your replay, the term of incidence is confusing, so please replace the the term ‘incidence’ in the abstract or use another term to avoid misunderstandings.

*DLG: In order to avoid this, the term high incidence has been changed by various cases.*

- As you agree in your replay, a low diversity usually indicates an invasion event. Therefore, please discuss this point more in detail in the discussion.
- *DLG: We have added your suggestion (low diversity could indicate an invasion event). It has been done in the area where we had discussed this matter (Line 212 in the current version).*
- Regarding the use of ITS-2, again, your data do not tell us anything about the origin of Bsal and are only valuable for the distribution of Bsal.
- *DLG: We are trying to show here the contrast between Bd and Bsal. We know that Bd has a high variability when sequenced in Okinawa (see for more Goka et al 2009, 2021). On the other hand, we have not detected any variability in the sequences obtained from Bsal in Okinawa. This situation and lack of variability among Bsal sequences could be interpreted as a difference in the time that both pathogens have been present in Okinawa, and I hope that you interpret our data in the same way.*
- Regarding your conclusions, again I think your data are not informative to say ‘this fungus has a long history in this region’.
  Additionally, the sentence ‘The found prevalence existing in our monitoring, 1.6%, is lower than in other studies in presumably Bsal native countries’ is incorrect because a proportion of 11/650 (your study) is not significantly lower than 33/1143 (China) nor 17/583 (Vietnam; Z=1.572, p=0.1160; Z=1.44, p=0.1499; respectively).
- *DLG: We have revised the Conclusion to address your points. Our results do not suggest, as you say, that this fungus has a long history in the area, but we have at least stated that it is highly compatible with that idea. For example, If you check all the studies that have been done about Bsal in Asia, (China, Vietnam, and Taiwan), you may notice that after the first detection carried out in these countries Bsal was not detected in the same localities or even to confirm the Bsal presence in the country was not possible again. However if you have any problem with this term, I will be more specific and being less assertive, rephrasing the sentence in this manner:* *“Our study confirms the presence of Batrachochytrium salamandrivorans in Japan, suggesting that this fungus may have a longer history in this region in comparison other Asian countries where recurrent detection has not been achieved, and in addition, it is not causing obvious mass mortalities as it does in other parts of the world”.*

*About the second part, the numbers of the prevalence, we have not mentioned any statistical term along the text. Your calculations are correct, but we are talking about the raw numbers, and we were not including the word significant, so our information about the prevalence is correct. In order to avoid any misleading information for the reader we have included between brackets “(But not significantly lower”) and “slightly” lower in the part that you have mentioned. Please see the text, line 238.*
